# Supplementary material for: Recreational screen time and adolescents’ school adjustment based on latent profile analysis: The mediating role of perceived physical health
Source: PLoS One. 2025 Sep 8;20(9):e0331584. doi: 10.1371/journal.pone.0331584 (PMC12416680; doi:10.1371/journal.pone.0331584)
Supplement: Attachment — Submitted filename: Response to reviewers.docx [file pone.0331584.s002.docx]

Dear Editor/Reviewer:

Thank you very much for your interest and recognition of this study and for giving us the opportunity to revise and improve **"Recreational screen time and adolescent school adjustment based on latent profile analysis: the mediating role of perceived physical health" (ID:PONE-D-25-21675)**. Your valuable suggestions have made an important contribution to the quality improvement of the manuscript, which we have taken seriously and have been substantially revised and improved. We sincerely hope that the manuscript will be published in **Plos One**.

This letter is followed by the editor/reviewers' comments and our point-by-point response to the reviewers' comments. Changes in the manuscript are marked using highlight mode. The revision was developed in consultation with all co-authors and each author approved the revision in its final form.

Thank you again for your time and advice.We look forward to hearing from you at your earliest convenience

Sincerely yours,

Jian Yang, Ph.D

**ACADEMIC EDITOR:**

**The different pattern observed for the "ambivalent" type regarding the mediating role of perceived physical health suggests an area for deeper investigation. The manuscript points out that recreational screen time did not predict school adjustment for this particular subgroup through the mediating effect of perceived physical health, and notes that the "physical and psychological developmental patterns and influencing factors of ambivalent school-adjusted adolescents need to be further explored". This highlights that while the study successfully identified this distinct subgroup, understanding the specific mechanisms affecting their school adjustment remains less clear, pointing towards a necessary direction for more nuanced future research specifically focused on this particular profile to uncover the unique factors at play. This isn't a flaw in the current study's execution but an acknowledged gap in understanding revealed by its findings, suggesting valuable avenues for future work that would improve upon the current study's scope in this specific area. Please, make a discussion by expressing these facts and ideas for future research.**

****Response:**Thank you for your thorough review of our paper and your valuable feedback. Your comments have been extremely helpful in further refining our research and improving the quality of the paper. In response to your suggestion regarding the study of the “Ambivalent Type” adolescent group, we have had in-depth discussions and made revisions. We hope the following explanations and additions can address your concerns.**

**In our study, we indeed found that recreational screen time significantly predicts the school adjustment of adolescents in the “Ideal Type” and “Growth Type” categories, with perceived physical health playing an intermediary role in this process. However, for the “Ambivalent Type” group, recreational screen time not only fails to directly predict their school adjustment but also cannot indirectly predict their school adjustment through perceived physical health as a mediating factor. This result highlights the particularities of the “Ambivalent Type” adolescent group in terms of school adjustment. Despite performing better in social capabilities such as interpersonal relationships, self-management, and academic skills compared to the “Growth Type”, the difficulties related to antisocial behavior may lead to different impacts on their school adjustment.**

**We speculate that for “Ambivalent Type” adolescents, the relationship between recreational screen time and school adjustment may not be as direct as in other groups. Due to their higher tendency towards antisocial behavior, the increase in recreational screen time may not promote school adjustment through improved physical health or emotional status. On the contrary, it may exacerbate negative emotions and behaviors. Therefore, future research should further explore the specific mechanisms of school adjustment in “Ambivalent Type” adolescents, particularly focusing on the roles of emotional regulation, behavioral control, and other factors.**

**We agree with your observation that our current understanding of the school adjustment mechanisms for the “Ambivalent Type” group remains limited. Further exploration in this area is crucial for refining the theoretical framework of adolescent school adjustment. Future studies should consider more psychosocial variables and integrate factors such as emotional regulation, behavioral control, and social support to gain a deeper understanding of the adaptation process for this group. As a result, we have expanded the discussion section of our paper based on your valuable suggestions. The additional content is as follows:**

It is worth mentioning that in this study, recreational screen time cannot predict the school adjustment of adolescents in the “Ambivalent Type” profile through perceived physical health. This is directly related to the fact that recreational screen time does not predict the school adjustment of adolescents in the “Ambivalent Type” profile. Specifically, the impact of excessive recreational screen time on the school adjustment of “Ambivalent Type” adolescents may not be solely reflected through changes in perceived physical health. The increase in recreational screen time may not indirectly promote school adjustment by improving their physical and mental health, and may even exacerbate negative behaviors and emotional issues. Conversely, the adaptation difficulties of this group may stem more from individual factors such as emotional regulation, social support systems, and behavioral control, which may not have as direct a relationship with recreational screen time as seen in other groups. Therefore, future research should further explore the unique mechanisms of school adjustment in “Ambivalent Type” adolescents. In addition to recreational screen time and perceived physical health, factors such as emotional regulation, behavioral control, and social support may have a more direct impact on their school adjustment. Interventions for this group may need to focus more on their emotional management and behavioral adjustments to help them better regulate their emotions, reduce antisocial behaviors, and improve school adjustment. Furthermore, future research should incorporate more variables to explore how these factors interact, in order to provide more precise intervention strategies for the “Ambivalent Type” adolescent group. This will help in understanding the school adjustment mechanisms of this group more comprehensively and provide theoretical foundations for improving their physical and mental development. ****(See lines 444-467)****

**In the third suggestion, “**Policy and Societal Level: Educational management institutions should implement more specific guidelines for adolescents' entertainment screen use, strengthening the awareness of “digital citizenship”. They should also encourage universities and research institutions to explore broader factors that contribute to improving school adjustment, especially for “Ambivalent Type” adolescents. Additionally, efforts should be made to promote collaboration among families, schools, and communities to build a support network for school adjustment in the context of digital media, enhancing adolescents' self-management and social adaptation abilities in a complex media ecology”. ****(**See lines 513-521**)****

**Thank you again for your valuable suggestions. We hope to make up for the deficiencies in the current research through more detailed discussions. We also look forward to your further opinions and guidance.**

**Journal Requirements:**

**When submitting your revision, we need you to address these additional requirements.**

1. **Please ensure that your manuscript meets PLOS ONE's style requirements, including those for file naming. The PLOS ONE style templates can be found at https://journals.plos.org/plosone/s/file?id=wjVg/PLOSOne_formatting_sample_main_body.pdf and** <https://journals.plos.org/plosone/s/file?id=ba62/PLOSOne_formatting_sample_title_authors_affiliations.pdf>

****Response:**Thank you very much for your suggestions. We apologize for the imperfections in the manuscript format that we submitted. We have made further corrections to the article's font, table format, page numbers, line numbers, and other details according to the requirements outlined in the PDF to ensure compliance with the journal's formatting guidelines. We kindly ask you to review and check it once again.**

2. **PLOS requires an ORCID iD for the corresponding author in Editorial Manager on papers submitted after December 6th, 2016. Please ensure that you have an ORCID iD and that it is validated in Editorial Manager. To do this, go to ‘Update my Information’ (in the upper left-hand corner of the main menu), and click on the Fetch/Validate link next to the ORCID field. This will take you to the ORCID site and allow you to create a new iD or authenticate a pre-existing iD in Editorial Manager.**

****Response**:Thank you for your valuable suggestion. Following your advice, we have registered for an ORCID ID and have verified the corresponding author’s ORCID ID in the Editorial Manager. ORCID ID：https://orcid.org/0009-0008-8533-7097**

**3. Thank you for stating the following financial disclosure:**

**MOE (Ministry of Education) Foundation on Humanities and Social Sciences [grant numbers 22YJA890032].**

**Please state what role the funders took in the study. If the funders had no role, please state: "The funders had no role in study design, data collection and analysis, decision to publish, or preparation of the manuscript."**

**If this statement is not correct you must amend it as needed.**

**Please include this amended Role of Funder statement in your cover letter; we will change the online submission form on your behalf.**

****Response**:**Thank you for your suggestion. We have provided a more detailed explanation of the role played by the funders as per your request. **“This study was supported by funding awarded to Professor Yang Jian from the MOE (Ministry of Education) Foundation on Humanities and Social Sciences [Grant Numbers 22YJA890032]. In his capacity as the funding recipient, Professor Yang Jian contributed to the study design, data collection and analysis, as well as preparation of the manuscript.”** Due to our unfamiliarity with the operating system, we have included this statement in the cover letter and kindly request the editor’s assistance in updating this information. We sincerely appreciate your help once again.

**4. We note that you have indicated that there are restrictions to data sharing for this study. For studies involving human research participant data or other sensitive data, we encourage authors to share de-identified or anonymized data. However, when data cannot be publicly shared for ethical reasons, we allow authors to make their data sets available upon request. For information on unacceptable data access restrictions, please see http://journals.plos.org/plosone/s/data-availability#loc-unacceptable-data-a**

****Response:****Thank you for your guidance regarding data sharing policies. We fully endorse PLOS ONE's commitment to advancing open science and have carefully reviewed the journal's specific provisions on restricted data access (available at: https://journals.plos.org/plosone/s/data-availability#loc-acceptable-data-access-restrictions).

The data utilized in this study originate from the Population Health Data Archive (PHDA) (Identifier: CSTR:17970.11.A0031.202107.209.V1.0). In compliance with the database's mandatory security protocols:

Researchers must complete registration and pass eligibility review to obtain access rights; Approved users may analyze data exclusively within a secure virtual sandbox environment; Technical restrictions prohibit copying, downloading, or exporting raw data; Only processed aggregated results may be transmitted to researchers via email

These safeguards are implemented by the data custodians to ensure statutory compliance for sensitive information protection.

To proactively address your requirements, we have:

Explicitly stated in our Data Availability Statement:

"Study data are subject to PHDA access protocols and require registration/approval (Access link: https://www.ncmi.cn/phda/dataDetails.do?id=CSTR:17970.11.A0031.202107.209.V1.0)."

This access model aligns with established practices in peer-reviewed publications such as:

Li et al. (2024) in BMC Psychology (DOI: 10.1186/s40359-024-01719-4)

Zhao et al. (2024) in Journal of Public Health (DOI: 10.1007/s10389-024-02316-w)

Should further refinements to our approach be needed, we would be pleased to implement specific recommendations. We sincerely appreciate your professional dedication to balancing scholarly standards with data security.

5. **Your abstract cannot contain citations. Please only include citations in the body text of the manuscript, and ensure that they remain in ascending numerical order on first mention.**

****Response:****Thank you for your reminder. We have thoroughly checked and revised the abstract section of the paper to ensure that the current version of the abstract does not contain any references to citations.

**6. Please update your submission to use the PLOS LaTeX template. The template and more information on our requirements for LaTeX submissions can be found at http://journals.plos.org/plosone/s/latex.**

****Response:**Thank you very much for your constructive feedback. Please allow me to express my sincere appreciation for your time and effort in handling our manuscript and reviewing it.**

**We fully understand and respect the journal's strict requirements regarding manuscript formatting, as outlined in the PLOS LaTeX template. This is important for ensuring the consistency and professionalism of the publication. However, we would like to openly share some technical difficulties we have encountered. During the writing phase, we primarily used Microsoft Word, which is the tool we are most familiar with. After receiving your request, we immediately began attempting to convert our manuscript into LaTeX format, following the guidelines provided by your journal (http://journals.plos.org/plosone/s/latex) and utilizing the official template (plos_latex_template.tex).**

**Unfortunately, despite our efforts, we encountered unforeseen technical obstacles during the conversion process, mainly due to our limited experience with LaTeX syntax and compilation procedures. These challenges were particularly noticeable when dealing with complex tables, ensuring accurate citation formatting, and overall compiling and debugging. We deeply regret that we were not able to meet the formatting requirements on the first submission.**

**Considering the valuable time of both you and the reviewers, we respectfully ask for your understanding and kind assistance. We sincerely inquire whether it would be possible to proceed with the review based on our current Word document, as the scientific content is finalized and fully meets the requirements. We recognize the importance of formatting and assure you that once the manuscript receives a principal acceptance, we will dedicate all necessary resources to strictly conform to the PLOS LaTeX template within the specified time frame. We will seek professional assistance or engage in further study to ensure a successful conversion. Alternatively, would it be possible to offer any additional support? If submission in LaTeX is indeed a prerequisite for the review process, would the journal’s editorial team be able to provide some basic conversion assistance or a more detailed troubleshooting guide? We would be happy to provide the complete Word document and related materials to facilitate any possible support.**

**We fully acknowledge that this situation may add additional consideration for you and the reviewers, for which we deeply apologize. It is certainly not our intention to disregard the journal’s requirements, but rather we have encountered technical challenges. We greatly value the opportunity to publish in PLOS ONE and are committed to doing everything necessary to ensure that the manuscript ultimately fully complies with all formatting standards.**

**Whatever your decision may be, we fully respect it and will take immediate action on the next steps. If leniency cannot be extended, we will double our efforts to overcome the technical difficulties and resubmit a version that fully complies with the LaTeX template as soon as possible.**

**Once again, thank you for your understanding, patience, and consideration of our manuscript. We look forward to your response.**

**Reviewer #1:**

**1.The Introduction should primarily provide background information, clearly identify the problem being addressed, and then outline the research objectives, questions, and structure of the paper. Research hypotheses should not be included in this section. It is recommended that the authors create a separate section for the research hypotheses and supplement this part with additional relevant literature.The authors are also advised to include a model diagram of the research hypotheses. This will help present the study’s hypotheses and theoretical framework in a clearer and more intuitive way.**

****Response:****Thank you for your valuable feedback and suggestions on our paper. We fully agree with and appreciate your input. Based on your comments, we have made corresponding revisions and improvements to the introduction, as well as added a research model diagram. Below is an outline of our adjustments:

First, we have clarified the research background and the problem. The introduction section now provides a detailed explanation of the definition of school adjustment, its importance in adolescent development, and the factors that influence school adjustment. This content offers readers a clear research background, helping them understand the core issue that this study aims to address: the relationship between recreational screen time and school adjustment in adolescents. We believe that sufficient background information lays a solid foundation for the subsequent research questions.

Second, based on your suggestion, we recognize that the research hypothesis should be a further inference based on existing literature, rather than a simple introduction to the theoretical background. Therefore, this part has been removed from the introduction. To improve the clarity of the article, we have listed the research hypotheses separately and provided more relevant literature to support them.

In this section, we have further strengthened the discussion on research related to recreational screen time, perceived physical health, and school adjustment. We supplemented the related literature, especially in the areas of the relationship between screen time, adolescent physical and mental health, academic performance, and school adjustment. More empirical studies and theoretical background have been added to enhance the integrity and logical flow of this section.

Lastly, regarding your point that "the introduction should clearly outline the structure of the paper," we have briefly introduced the structure of the paper at the end of the introduction. We have clarified how this study will use Latent Profile Analysis (LPA) to more precisely analyze the impact of recreational screen time on school adjustment and its mediating role through perceived physical health. Additionally, we have added a research model diagram to clearly illustrate the research hypotheses, as shown in Fig. 1. **(See lines 19-134)**

We believe these revisions address your concerns and make the introduction more aligned with academic paper standards. Should you have any further suggestions or feedback, we would be grateful and will continue to make improvements.

Once again, thank you for your support and guidance in our research!

**Fig.1 Research model**

**2.By dividing the adolescent sample into three groups and using multiple regression to test both the direct effect of leisure screen time on school adjustment and the mediating role of perceived physical health, the authors offer initial insights into group differences. However, this approach does not account for self-report measurement error and treats latent profile classification and path analysis as separate, overlooking classification uncertainty and the hierarchical structure of the data. It is recommended that the authors use structural equation modeling (SEM) to systematically examine the measurement models and path relationships among the key latent variables—leisure screen time, school adjustment, and perceived physical health. Afterwards, multiple regression analysis can be used to reveal differences in performance among the latent groups.**

****Response:****We sincerely appreciate your detailed review and valuable suggestions. Your comments on self-report measurement errors, the integration of Latent Profile Analysis (LPA) with path analysis, and data hierarchy issues have provided us with valuable insights and have been crucial in further improving our research methodology and analytical framework.

In this study, we followed a chain of logic: group division → variable relationships → mechanism exploration. First, we used LPA to stratify the dependent variable, "school adjustment." The results indicated that dividing the adolescent population into three profiles was most appropriate. We also retained the total sample data as a control group, forming four distinct data groups. Next, we employed Ordinary Least Squares (OLS) regression analysis to build four models, examining the relationship between the independent variable (recreational screen time) and the dependent variable (school adjustment). Upon finding a significant relationship between these two variables, we further investigated the mediating role of perceived physical health in this relationship.

In response to your concerns regarding measurement errors and analysis methods, we first constructed a Structural Equation Model (SEM) before conducting the mediation analysis, in order to more comprehensively and systematically examine the measurement model and path relationships between recreational screen time, perceived physical health, and school adjustment. Given that we have already presented the correlation analysis results in the multiple linear regression and to avoid repetition of numerical details, we have not separately displayed the SEM path diagram. Instead, we have paid particular attention to the model fit indices, such as CMIN/DF, CFI, TLI, GFI, and RMSEA, to ensure that the model fits the data well, thereby enhancing the robustness of our analysis. By conducting a fit test of the SEM model, we aim to address your concerns regarding the combination of LPA and path analysis, further enhancing the reasonableness and transparency of our analysis.

The specific modifications are as follows:

To systematically test the model fit between the research model and the observed data, Structural Equation Modeling (SEM) was conducted using AMOS. After incorporating the independent variable, mediating variable, dependent variable, and control variables, it was found that Model 1, Model 3, and Model 4 had good model fit indices (although CMIN/DF was slightly large, the results were still acceptable considering the large sample size). However, since the TLI value of Model 2 was much lower than 0.9, its fit indices were not ideal. Detailed results are shown in Table 6.

**Table 6 Model Fitting Indicators**

| Model type | CMIN/DF | CFI | TLI | GFI | RMSEA |
| --- | --- | --- | --- | --- | --- |
| Model 1 | 4.976 | 0.979 | 0.918 | 0.996 | 0.035 |
| Model 2 | 4.719 | 0.944 | 0.780 | 0.992 | 0.051 |
| Model 3 | 6.942 | 0.990 | 0.959 | 0.998 | 0.027 |
| Model 4 | 11.798 | 0.990 | 0.959 | 0.998 | 0.029 |

**Note: Model 1 represents the “Growth Type”, Model 2 represents the “Ambivalent Type”, Model 3 represents the “Ideal Type”, and Model 4 represents the overall type.**

Subsequently, the Bootstrap extraction was set to 5000 times in SPSS 26.0 using the Process plugin for mediation effect testing. The results are shown in Table 7. The study found that recreational screen time can predict school adjustment in Models 1, 3, and 4 through the mediation effect of perceived physical health (95% CI: -0.044, -0.012; 95% CI: -0.060, -0.038; 95% CI: -0.179, -0.148), but it cannot predict school adjustment in Model 2 through perceived physical health (95% CI: -0.028, 0.018).**(See lines 305-321)**

We believe these adjustments will enhance the rigor of the research and fully take into account your opinions and suggestions. Thank you for providing such valuable feedback, and we look forward to your further guidance and comments.

**3.In the references section, some journal names are abbreviated while others are written in full. The authors are advised to standardize the journal name formatting according to the journal’s citation guidelines to enhance the consistency and professionalism of the references.**

****Response:****Thank you for your professional suggestions regarding the reference section. We fully agree with your feedback. The issue arose from our use of reference management software, and unfortunately, due to an oversight in our handling, this error occurred. We have now thoroughly revised and improved the reference section according to the journal's requirements, with particular attention to the accuracy of journal names, ensuring they are all listed in their full titles.

The modified references are as follows:

Dawes M. Early adolescents’ social goals and school adjustment. Social Psychology of Education. 2017;20: 299–328. doi:10.1007/s11218-017-9380-3

Liu M, Wu L, Yao S. Dose–response association of screen time-based sedentary behaviour in children and adolescents and depression: a meta-analysis of observational studies. British Journal of Sports Medicine. 2016;50: 1252–1258. doi:10.1136/bjsports-2015-095084

Chaput J-P, Willumsen J, Bull F, Chou R, Ekelund U, Firth J, et al. 2020 WHO guidelines on physical activity and sedentary behaviour for children and adolescents aged 5–17 years: summary of the evidence. International Journal of Behavioral Nutrition and Physical Activity. 2020;17: 141. doi:10.1186/s12966-020-01037-z

Tremblay MS, Carson V, Chaput J-P, Connor Gorber S, Dinh T, Duggan M, et al. Canadian 24-Hour Movement Guidelines for Children and Youth: An Integration of Physical Activity, Sedentary Behaviour, and Sleep. Applied Physiology, Nutrition, and Metabolism. 2016;41: S311–S327. doi:10.1139/apnm-2016-0151

Legkauskas V, Steponavičiūtė-Kupčinskė I. Links between in-class use of social media and school adjustment of high-school pupils. Education and Information Technologies. 2021;26: 2853–2861. doi:10.1007/s10639-020-10389-9

Choi M, Park S. The Mediating Effects of Academic Performance between Screen Time, Executive Function Difficulty and School Adjustment. Comprehensive Child and Adolescent Nursing-Building Evidence for Practice. 2020;43: 334–347. doi:10.1080/24694193.2019.1675805

Li L, Zhang Y, Fan M, Cao B. Sleep and mental health among Chinese adolescents: the chain-mediating role of physical health perception and school adjustment. BMC Psychology. 2024;12: 228. doi:10.1186/s40359-024-01719-4

Fryer LK. (Latent) transitions to learning at university: A latent profile transition analysis of first-year Japanese students. Higher Education. 2017;73: 519–537. doi:10.1007/s10734-016-0094-9

Elbilgahy AA, Sweelam RK, Eltaib FA, Bayomy HE, Elwasefy SA. Effects of Electronic Devices and Internet Addiction on Sleep and Academic Performance Among Female Egyptian and Saudi Nursing Students: A Comparative Study. Sage Open Nursing. 2021;7: 23779608211055614. doi:10.1177/23779608211055614

Sümen A, Evgin D. Social Media Addiction in High School Students: A Cross-Sectional Study Examining Its Relationship with Sleep Quality and Psychological Problems. Child Indicators Research. 2021;14: 2265–2283. doi:10.1007/s12187-021-09838-9

Paradis AD, Koenen KC, Fitzmaurice GM, Buka SL. Impact of persistent and adolescent-limited antisocial behaviour on adult health outcomes. Journal of Epidemiolgy and Community Health. 2016;70: 1004–1010. doi:10.1136/jech-2014-205140

**4.There are also some minor errors in the manuscript. The authors are encouraged to carefully review the entire paper and make necessary corrections to further improve its clarity and overall quality.**

****Response:****First of all, we apologize for the minor errors in the manuscript and appreciate your patience and attention to detail during the review process. Following your suggestions, we sought the assistance of doctoral students with a strong command of English to carry out the necessary corrections to the manuscript. We have addressed issues related to grammar, technical terminology, and other details. We believe the quality of the revised manuscript has significantly improved. Once again, we thank you for your patience and valuable feedback.

**Reviewer #2:**

**1.Many thanks for your invitation to review the manuscript titled "Recreational screen time and adolescent school adjustment based on latent profile analysis: the mediating role of perceived physical health." Overall, the manuscript topic is interesting and presents valuable findings for the adolescent group. However, the manuscript requires minor revisions before its acceptance.
In the result section, it is stated that "overall sample category should not be less than 5% [32]", the authors may also add a primary source reference with the existing one.
**Response:****Thank you very much for your interest in the topic of this study and for your valuable suggestions. We fully agree with the suggestions you provided and have added the key references as per your advice. **(See lines 219)**

**References**

**Marsh HW, Lüdtke ,Oliver, Trautwein ,Ulrich, and Morin AJS. Classical Latent Profile Analysis of Academic Self-Concept Dimensions: Synergy of Person- and Variable-Centered Approaches to Theoretical Models of Self-Concept. Structural Equation Modeling: A Multidisciplinary Journal. 2009;16: 191–225. doi:10.1080/10705510902751010**

**2.Overall, minor grammatical mistakes need to be addressed.
**Response:****Thank you very much for your appreciation and praise for this article. At the same time, we would like to apologize for the grammatical errors. Subsequently, we consulted a PhD student with a strong foundation in English to carefully revise and improve the grammar of the article. We believe that, with these further revisions, the language of the article has been significantly enhanced.

**3.The conclusion is way too short. The authors could provide additional recommendations to support the target group who might benefit from the study's findings, such as the study's potential for home environment approaches or school administration actions.**

****Response:****Thank you very much for your constructive suggestions. We have realized that the conclusion was too brief. Based on your advice, we have specifically proposed research recommendations according to the results of the study. We discussed the groups that might benefit from the findings and provided targeted recommendations at the levels of family, school, and other relevant contexts.

The revised conclusion and recommendations are as follows:

Adolescent school adjustment exhibits heterogeneity and can be categorized into three types: “Ideal Type”, “Growth Type”, and “Ambivalent Type”. Recreational screen time can significantly negatively predict the level of school adjustment and the probability of belonging to different categories. Specifically, the longer the duration of entertainment screen use, the more likely individuals are to be classified as “Growth Type” or “Ambivalent Type”, moving further away from the “Ideal Type”. Furthermore, recreational screen time indirectly influences school adjustment performance through the mediating mechanism of perceived physical health. Specifically, excessive entertainment screen use weakens adolescents' subjective evaluations of their health, reducing their psychological vitality and physical confidence, which negatively affects their self-adjustment and social interaction abilities in the school environment. This study not only expands the theoretical perspective of adolescent school adjustment classification but also reveals the impact pathways of digital lifestyles on school adjustment. Based on these findings, the following recommendations are proposed:

Family Level: Parents are encouraged to strengthen the daily management of adolescents' entertainment screen use, especially paying attention to the duration, usage context, and content type. They should help adolescents develop good media usage habits and health awareness. Additionally, parents should create more opportunities for parent-child interaction, outdoor exercise, and structured learning, in order to increase chances for positive feedback in real life and promote the development of social skills.

School Level: Schools can use psychological assessments and behavioral observations to identify “Growth Type” and “Ambivalent Type” students. Tailored intervention programs, such as social skills training, conflict management courses, and emotional regulation training, should be implemented to enhance students' adaptability. Schools should also emphasize health literacy and media literacy education, helping students establish an integrated cognitive system encompassing physical, psychological, and social health, to resist the adverse effects of excessive screen use on health and interpersonal functioning.

Policy and Societal Level: Educational management institutions should implement more specific guidelines for adolescents' entertainment screen use, strengthening the awareness of “digital citizenship”. They should also encourage universities and research institutions to explore broader factors that contribute to improving school adjustment, especially for “Ambivalent Type” adolescents. Additionally, efforts should be made to promote collaboration among families, schools, and communities to build a support network for school adjustment in the context of digital media, enhancing adolescents' self-management and social adaptation abilities in a complex media ecology.**(See lines 483-521)**

*Once again, we sincerely thank the editors and reviewers of PLOS One for their valuable suggestions.*
